# E3-ubiquitin ligases and recent progress in osteoimmunology

**DOI:** 10.3389/fimmu.2023.1120710

**Published:** 2023-02-23

**Authors:** Yosuke Asano, Yoshinori Matsumoto, Jun Wada, Robert Rottapel

**Affiliations:** ^1^ Department of Nephrology, Rheumatology, Endocrinology and Metabolism, Okayama University Faculty of Medicine, Dentistry and Pharmaceutical Sciences, Okayama, Japan; ^2^ Princess Margaret Cancer Center, University Health Network, University of Toronto, Toronto, ON, Canada; ^3^ Department of Medicine, University of Toronto, Toronto, ON, Canada; ^4^ Department of Medical Biophysics, University of Toronto, Toronto, ON, Canada; ^5^ Department of Immunology, University of Toronto, Toronto, ON, Canada; ^6^ Division of Rheumatology, St. Michael’s Hospital, Toronto, ON, Canada

**Keywords:** E3-ubiquitin ligases, ubiquitylation, proteasomal degradation, osteoimmunology, cherubism

## Abstract

Ubiquitin-mediated proteasomal degradation is a post-transcriptional protein modification that is comprised of various components including the 76-amino acid protein ubiquitin (Ub), Ub-activating enzyme (E1), Ub-conjugating enzyme (E2), ubiquitin ligase (E3), deubiquitinating enzyme (DUB) and proteasome. We and others have recently provided genetic evidence showing that E3-ubiquitin ligases are associated with bone metabolism, the immune system and inflammation through ubiquitylation and subsequent degradation of their substrates. Dysregulation of the E3-ubiquitin ligase RNF146-mediated degradation of the adaptor protein 3BP2 (SH3 domain-binding protein 2) causes cherubism, an autosomal dominant disorder associated with severe inflammatory craniofacial dysmorphia syndrome in children. In this review, on the basis of our discoveries in cherubism, we summarize new insights into the roles of E3-ubiquitin ligases in the development of human disorders caused by an abnormal osteoimmune system by highlighting recent genetic evidence obtained in both human and animal model studies.

## 1 Introduction

**Figure 1:**
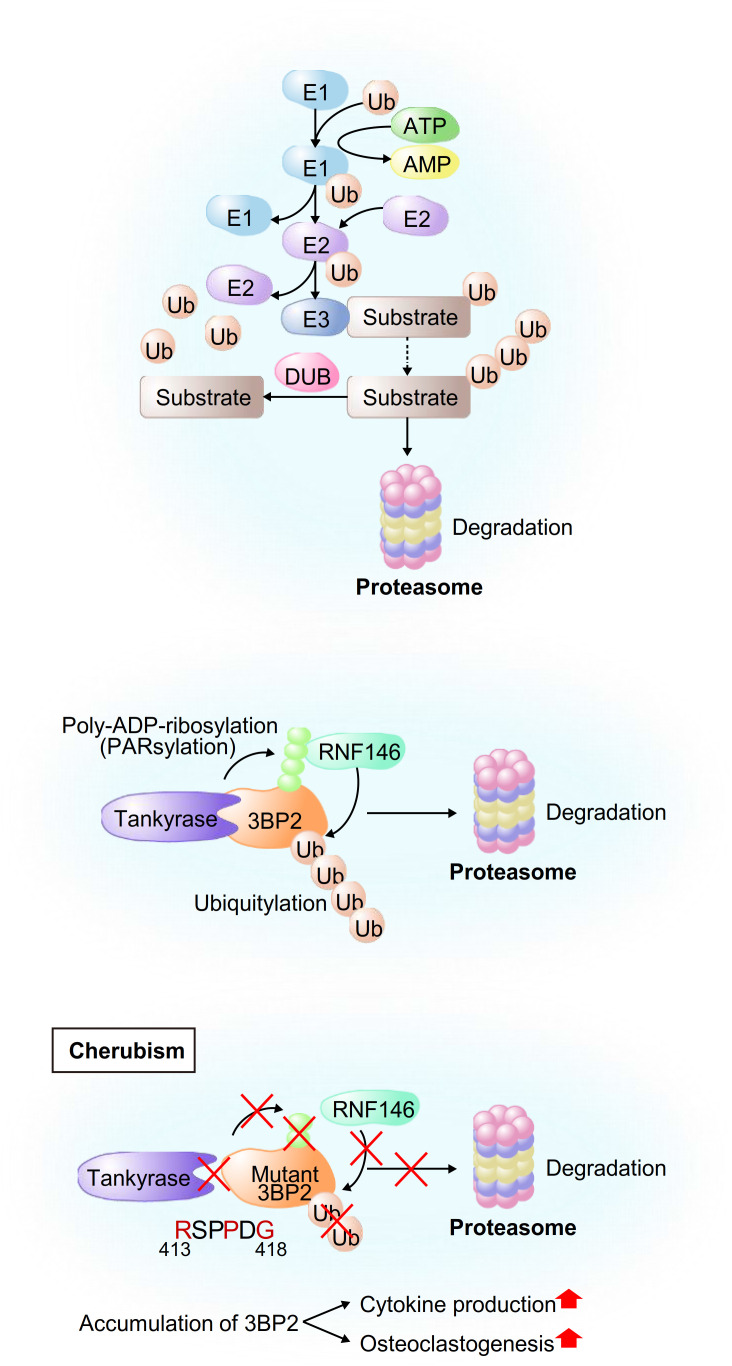
Schematic model of ubiquitylation of general proteins and 3BP2. (Upper) Schematic model of proteasomal degradation mediated by E3-ubiquitin ligases. First, E1 activates ubiquitin and forms an E1-ubiquitin intermediate. Ubiquitin is then transferred from E1 to E2, leading to the formation of an E2-ubiquitin intermediate. Finally, E3 recognizes its substrate proteins and the E2-ubiquitin intermediate, resulting in the formation of a protein complex and transference of the activated ubiquitin from E2 to the substrates. (Middle and Bottom) Schematic model of the development of cherubism. Tankyrase-mediated PARsylation of 3BP2 creates a recognition site for RNF146, leading to ubiquitylation and subsequent proteasomal degradation of 3BP2 (middle). Cherubism mutations uncouple 3BP2 from Tankyrase, which results in impairment of RNF146-mediated ubiquitylation and subsequent stabilization of 3BP2 in macrophages, leading to activation of cytokine production and osteoclastogenesis (bottom).

Ubiquitin-mediated proteasomal degradation is a post-transcriptional protein modification that is comprised of various components including the 76-amino acid protein ubiquitin (Ub), Ub-activating enzyme (E1), Ub-conjugating enzyme (E2), ubiquitin ligase (E3), deubiquitinating enzyme (DUB) and proteasome. E1 activates ubiquitin and forms an E1-ubiquitin intermediate, and ubiquitin is transferred from E1 to E2, leading to the formation of an E2-ubiquitin intermediate (1–3). Then E3 recognizes its substrate proteins and the E2-ubiquitin intermediate, resulting in the formation of a protein complex and transference of the activated ubiquitin from E2 to most often a lysine residue in the substrates (4). Ubiquitin has seven lysine residues, including K6, K11, K27, K29, K33, K48 and K63, that are used as attachment sites for subsequent Ub proteins (1, 2, 4), while K48-linked chains are the most abundant linkages for polyubiquitylation. This process is repeated to form a polyubiquitin chain, and ubiquitin-tagged proteins are recognized and degraded into small fragments by the 26S proteasome. Protein ubiquitylation is reversed by DUBs that hydrolyze the peptide bonds linking the substrates to ubiquitin (1, 2, 5) (**Figure 1**, top). More than 600 E3-ubiquitin ligases have been identified in humans (6) and they are classified into four major groups on the basis of their structures: HECT (homologous to E6-AP carboxyl terminus) type, RING (really interesting new gene)-finger type, U-box type and RBR (RING-between RING-RING) type (7, 8).

We have extensively investigated the roles of E3-ubiquitin ligases in bone metabolism, the immune system and inflammation through ubiquitylation and subsequent degradation of their substrates. The adaptor protein 3BP2 (SH3 domain-binding protein 2) nucleates a signaling complex including ABL, SRC, VAV and SYK and enforces an open active configuration of these proteins, leading to their kinase activation. Gain-of-function missense mutations in the *SH3BP2* gene cause cherubism, an autosomal dominant disorder associated with severe inflammatory craniofacial dysmorphia syndrome in children (9, 10). Prof. Robert Rottapel’s group at the University of Toronto have provided a mechanistic understanding of cherubism by showing that impairment of ubiquitylation of the cherubism mutant 3BP2 leads to accumulation of 3BP2 and subsequent activation of osteoclastogenesis and cytokine production in macrophages (11, 12).

On the basis of our discoveries in cherubism, we have provided further genetic and mechanistic evidence showing that E3-ubiquitin ligase-mediated protein degradation of their substrates is associated with various human disorders caused by an abnormal osteoimmune system.

In this review, we summarize new insights into the roles of E3-ubiquitin ligases in the development of human disorders caused by an abnormal osteoimmune system by highlighting recently reported genetic studies that have provided mechanistic evidence.

## 2 HECT-domain E3-ubiquitin ligases and human disorders

**Figure 2:**
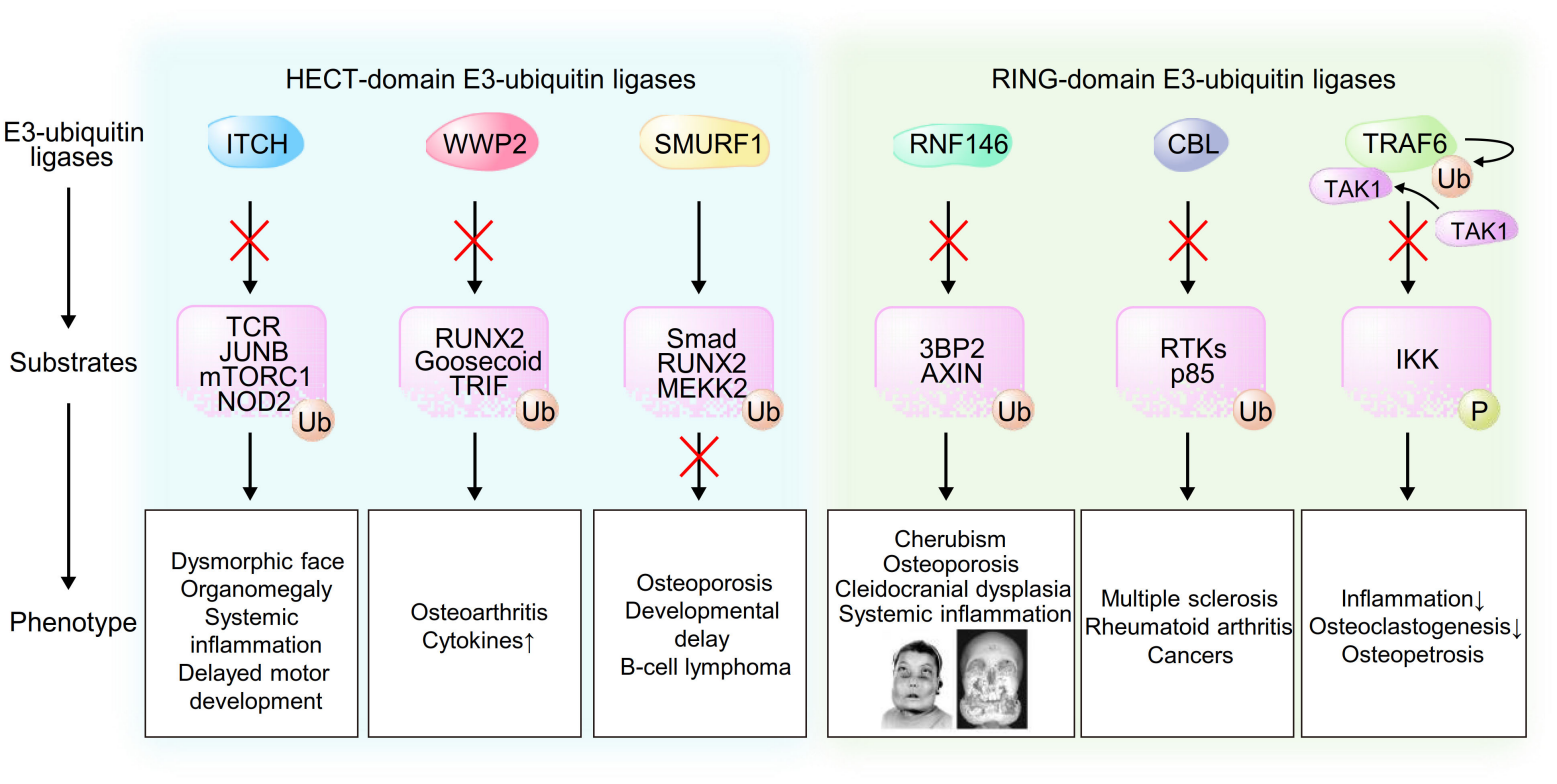
Schematic model of the associations between E3-ubiquitin ligases, their substrates and phenotype caused by dysregulation of these pathways. Pictures of an individual reproduced from Ueki et al. *Nat Genet*. 28(2):125-126, 2001 (9).

Recent studies have provided genetic evidence linking HECT-domain E3-ubiquitin ligases, which contain an N-terminal C2 domain, a WW domain and a C-terminal catalytic HECT domain, to the pathogenesis of human disorders. ITCH was originally identified in the mouse *agouti* locus, in which mutations lead to characteristic coat color changes (13). In humans, Lohr et al. reported the first pathogenic mutation in the *ITCH* gene that resulted in ITCH deficiency in ten Amish children with multisystem autoimmune disease and developmental abnormalities (14). After various examinations that failed to reveal a diagnosis, they performed single-nucleotide polymorphism autozygosity mapping and identified a large homozygous block in chromosome 20q11. They further found a pathogenic truncating homozygous mutation in the *ITCH* gene in all of the affected children. Characteristic clinical features were dysmorphic faces, multiple organomegaly of the lung, liver and gut with inflammatory cell infiltration and delayed motor development, indicating that human ITCH deficiency leads to a complex phenotype affecting physical growth, craniofacial morphology, muscle development and immune function. Three children died due to respiratory failure caused by cellular nonspecific interstitial pneumonitis. Mouse studies and *in vitro* studies have provided mechanistic evidence of these clinical features. Mutations of Itch cause a fatal autoimmune disease characterized by histiocyte and lymphocyte infiltration of the lungs, liver, kidney and heart in mice that shows a phenotypic similarity to human diseases (15). Both antigen processing and T-cell anergy are abnormal (16), and multiple organs are infiltrated by lymphocytes, particularly autoreactive B cells, leading to fatal lung disease early in life. Ubiquitylation of the TCR (T-cell receptor) results in its downregulation (17), and downstream signaling is also altered by ubiquitylation of JUNB, which may inhibit IL-2 production and T-cell proliferation (15). Itch deficiency results in loss of tolerance to self-antigens and autoreactive T/B cells (18). ITCH is also a negative regulator of the BCR (B-cell receptor) signaling pathway possibly through ubiquitylation and regulation of components of the mTORC1 complex that are key contributors to glycolysis in B cells and maintenance of germinal centers (19). Itch-deficient B cells have been shown to exhibit increased survival and proliferation through activation of mTORC1-mediated glycolysis (19). Detailed studies on immunological function, B-cell regulation and autoimmunity are currently underway. In addition to immune cells, ITCH is expressed in various tissues including the gut, pancreas, nerves and lymphoid tissues. ITCH might regulate NOD2 signaling (20), which could explain the bowel inflammation observed in these patients with ITCH deficiency since NOD is associated with activation of the innate immune system and its mutations have been identified in patients with Crohn’s disease (21). Craniofacial dysmorphic phenotype is associated with other osteoimmunological roles of ITCH in nonhematopoietic cells, possibly cells of mesenchymal origin. ITCH thus has critical functions in the maintenance of homeostasis in different physiologic states (**Figure 2**).

WWP2, which was originally identified as a protein binding to atrophin-1 from yeast two-hybrid screening and *in vitro* binding analysis and was named atrophin-1 interacting protein 2 (AIP2) (22), might be involved in the pathogenesis of several human disorders on the basis of results of previous *in vivo* studies. Gao et al. reported that WWP2 was identified from 187 genetic variants as a susceptible gene in osteoarthritis (OA) from a GWAS (genome-wide association study) (23). WWP2 is abundantly expressed in articular cartilage, and WWP2 expression level is decreased in human OA cartilage (24). Mice lacking WWP2 exhibit aggravated spontaneous and surgically induced OA since WWP2 protects cartilage through ubiquitylation and degradation of its substrate RUNX2 (runt-related transcription factor 2), which induces ADAMTS5 and subsequent cartilage degradation (24). WWP2 might also be associated with the development of congenital craniofacial anomalies (CFA) since WWP2-deficient mice display craniofacial malformation (25). In this regard, WWP2 interacts with and ubiquitylates the paired-like homeobox transcription factor Gsc (Goosecoid), leading to its transcriptional activation of Sox6, which plays an important role in craniofacial development. In addition to skeletal formation, WWP2 is associated with the innate immune system through regulation of the Toll-like receptor (TLR) signaling pathway. Upon TLR3 activation by viral double-stranded RNA, WWP2 mediates K48-linked ubiquitylation and degradation of TRIF (TIR-domain-containing adapter-inducing interferon-β), which is required for TLR3-mediated NF-κB and IRF3 (interferon regulatory factor 3) activation, leading to induction of proinflammatory cytokines and type I interferons (26). These findings indicate that loss-of-function mutations in the *WWP2* are associated with human skeletal and inflammatory disorders (**Figure 2**).

A number of *in vivo* and *in vitro* studies have shown associations of SMURF1 (Smad Ubiquitin Regulatory Factor-1) with osteoblast function and response to BMPs (bone morphogenetic proteins) (27, 28), and genetic variants in the genes encoding SMURF1-related proteins have been implicated in the risk of osteoporosis by hypothesis-free GWAS (29). Al−Rawi et al. reported the first case of a microduplication in the *SMURF1* gene in a 10-year-old girl suffering from two leg fractures with osteoporosis, severe developmental delay, infantile seizures and B-cell lymphoma (30). During the process of osteoblastogenesis, BMPs bind to homomeric type II receptor, which phosphorylates a glycine-serine-rich domain in homomeric type I receptor, leading to induction of signal transduction. The phosphorylated complex of receptor-regulated SMADs (R-Smad; Smad1/5/8) and Co-Smad (Smad4) translocates into the nucleus and binds to the promoter regions of target genes including the osteoblastogenic master transcription factor RUNX2 and Osterix, leading to osteoblast differentiation and maturation. Additionally, the binding of BMPs to the receptors leads to phosphorylation of mitogen-activated protein kinase kinase kinase 2 (MEKK2) and subsequent activation of the c-Jun N-terminal kinase (JNK) signaling pathway, leading to osteoblast activation and increased response to BMPs (31). In bone metabolism, SMURF1 inhibits R-Smad/Co-Smad complex nuclear translocation and directly ubiquitylates and degrades RUNX2, leading to suppression of osteoblastogenesis. Additionally, SMURF1 ubiquitylates and regulates MEKK2, resulting in inhibition of JNK activation and subsequent suppression of osteoblast activity and response to BMPs (28, 30). Consistent with these molecular mechanisms, clinical features in the reported child with osteoporosis and bone fractures showed phenotypic similarity to those observed in mice with Smurf1 mutations. In addition to its roles in skeletal development, SMURF1 may function as an oncoprotein through regulation of the levels of the tumor suppressor RhoB, leading to promotion of tumor metastasis and initiation (32), which may provide the reason why B-cell lymphoma developed in the girl (**Figure 2**).

## 3 RING-domain E3-ubiquitin ligases and human disorders

CBL (Casitas B-lineage lymphoma proto-oncogene) and CBL-B, a member of the CBL family of proteins including CBL, CBL-B and CBL-C, are RING-domain E3-ubiquitin ligases that ubiquitylate receptor tyrosine kinases (RTKs) for degradation as enzymes (33) and activate several signaling pathways through protein-protein interaction as adaptor proteins (34, 35). A previous GWAS and a previous EWAS (epigenome-wide association study) showed a genetic link between CBL and several human inflammatory disorders such as MS (multiple sclerosis) (36) and RA (rheumatoid arthritis) (37). CBL-B has been reported to ubiquitylate the regulatory p85 subunit of PI3K that results in inhibition of the recruitment of PI3K to CD28 upon activation of the costimulatory pathway rather than its degradation, leading to inhibition of T-cell activation (38). The pathogenic single nucleotide polymorphism of CBL-B in patients with MS is associated with reduction of CBL-B expression levels and induction of CD4+ T-cell proliferation mediated by type I IFNs (39). Additionally, loss-of-function mutations in the CBL gene are associated with the development of cancers since CBL has tumor suppressor functions through both ubiquitylation and degradation of RTKs and inhibition of the PI3K signaling pathway (37), indicating that CBL or CBL-B mutations might be involved in the pathogenesis of autoreactive inflammatory disorders and cancers in humans (**Figure 2**).

In addition to CBL family proteins, several genetic studies have revealed multiple risk alleles for autoantibody-positive RA within the *MHC* region, a *PTPN22* missense allele and risk alleles in other loci. Raychaudhuri et al. systematically examined 370 SNPs from 179 independent loci with *p*<0.001 in a published meta-analysis of an RA GWAS of 3,393 cases and 12,462 controls and identified *TRAF6/RAG1* as one of the true RA risk alleles (40). TRAF6 is a member of the TNF receptor associated factor (TRAF) protein family in which each protein contains an N-terminal RING domain, zinc-finger motifs, a central coiled-coil region and a highly conserved C-terminal domain and mediates signaling from the TNF receptor superfamily and Toll/IL-1 family. In the IL-1 signaling pathway, TRAF6 induces K-63-linked auto-ubiquitylation after its oligomerization, which results in the recruitment and activation of TAK1 (transforming growth factor β-activated kinase 1), leading to phosphorylation of IKK (IκB kinase) and subsequent activation of NF-κB and cytokine production. In addition to cytokine production, RANKL-induced signaling in macrophages and osteoclastogenesis are also controlled by TRAF6 since TRAF6-deficient mice display osteopetrosis due to an osteoclast defect (41) (**Figure 2**).

In addition to the roles of these RING-domain E3-ubiquitin ligases in the development of human disorders, we have uncovered another genetic link between RNF146 (Ring Finger Protein 146) and the human hereditary syndrome cherubism.

## 4 Newly identified roles of RNF146 in the osteoimmune system on the basis of discoveries in cherubism

### 4.1 Impairment of 3BP2 ubiquitylation by RNF146 causes cherubism

We have investigated the roles of the RING-domain E3-ubiquitin ligase RNF146, which contains a WWE domain and a RING domain (42, 43), in the osteoimmune system. RNF146 recognizes poly-ADP-ribosylated (PARsylated) proteins that are catalyzed by Tankyrase, a member of the PARP (Poly(ADP-ribose) polymerase) family, through its WWE domain, resulting in its structural change and subsequent activation of the RING domain. Then RNF146 induces K48-linked polyubiquitylation and subsequent degradation of its substrates by the 26S proteasome (43, 44). 3BP2 (SH3 domain-binding protein 2), which was originally identified as a binding protein for the ABL kinase (Abelson murine leukemia viral oncogene homolog 1) (45, 46), is one of the identified substrates that are regulated by RNF146-mediated ubiquitylation (**Figure 1**, middle). It has been reported that single missense mutations in the *SH3BP2* gene cause cherubism, a rare hereditary syndrome associated with severe craniofacial developmental defects in children (9, 47). Prof. Robert Rottapel’s group at the University of Toronto reported that cherubism mutations uncouple 3BP2 from Tankyrase, which results in impairment of RNF146-mediated ubiquitylation and subsequent stabilization of 3BP2 in macrophages, leading to activation of SYK and SRC kinases and hyperosteoclastogenesis (11, 12) (**Figure 1**, bottom). In addition to the roles of 3BP2 in osteoclastogenesis, 3BP2 is also required for osteoblastogenesis since *Sh3bp2^-/-^* mice display osteoporosis due to defective osteoblastogenesis (48). We have shown that 3BP2-mediated ABL kinase activation potentiates the formation of a transcriptional complex of the osteoblastogenic master transcription factor RUNX2 and TAZ (transcriptional co-activator with PDZ-binding motif), leading to phosphorylation and activation of RUNX2 and subsequent enhancement of osteoblastogenesis (49). ABL and TAZ reciprocally stabilize each other through suppression of the respective E3-ubiquitin ligases SMURF1 and β-TrCP. Similarly, we showed that ABL-mediated RUNX2 phosphorylation is also required for breast cancer invasion through an increase of *MMP13* transcripts that is independent of TAZ-mediated RUNX2 activation (50).

### 4.2 Impairment of RNF146-mediated ubiquitylation in bone cells causes human skeletal disorders

On the basis of our discoveries in cherubism, we have further investigated the genetic link between RNF146 and the osteoimmune system through generation of conditional knockout mice in which endogenous *Rnf146* is deleted in myeloid cells (*Rnf146^fl/fl^ LysM-Cre* (+)) or osteoblasts (*Rnf146^fl/fl^ Osterix-Cre* (+)). We showed that RANKL represses *RNF146* transcripts through activation of NF-κB and subsequent inhibition of the *RNF146* promoter (51). Repression of RNF146 by RANKL results in stabilization of its substrates 3BP2 and AXIN1, which triggers SRC activation and β-catenin attenuation, respectively, both of which are required to execute the osteoclast developmental program (11, 52–54). Consistently, we showed that dysfunction of the RNF146-mediated 3BP2 degradation program in *Rnf146^fl/fl^ LysM-Cre* (+) mice results in enhanced osteoclastogenesis and bone loss. Additionally, depletion of RNF146 leads to hypersensitivity to LPS-induced TNF-α production *in vivo*, indicating that RNF146 acts as an inhibitory switch controlling osteoclastogenesis and cytokine production that could be a control point underlying the pathogenesis of chronic inflammatory diseases (51). In addition to the roles of RNF146 in osteoclastogenesis and cytokine production, we have provided further genetic evidence showing that mice lacking RNF146 in osteoblasts show phenotypic similarities to cleidocranial dysplasia (CCD) (55), an autosomal dominant human disorder characterized by abnormal bone development mainly due to defective intramembranous bone formation by osteoblasts (56, 57). We showed that loss of RNF146 in osteoblasts stabilizes AXIN1, which results in inhibition of Wnt3a-induced β-catenin activation and reduced *Fgf18* expression. FGF18 induces TAZ expression required for osteoblast proliferation and differentiation through activation of TEAD and RUNX2 transcription factors, respectively. These findings indicate that dysfunction of RNF146 could be the pathogenesis of CCD in addition to known mutations in a single allele of *RUNX2* (55, 58–63) (**Figure 2**).

## 5 Discussion and future perspectives

In this review, we have summarized the roles of E3-ubiquitin ligases in the development of human disorders caused by an abnormal osteoimmune system. On the basis of our discoveries in cherubism, we and others have further investigated and provided evidence that links E3-ubiquitin ligases to autoimmune/autoinflammatory disorders. We have shown that mice lacking Tankyrase in myeloid cells develop severe systemic inflammation with elevated inflammatory cytokine production through the impairment of RNF146-medaited 3BP2 degradation (64). An increased level of 3BP2 in macrophages results in tyrosine phosphorylation and activation of TLR2, and TLR2 (Y647) phosphorylation within the TIR domain by SRC and SYK is essential for TLR2 stabilization and signaling. In the myeloid cell lineage, 3BP2 is also required for G protein-coupled receptor-mediated neutrophil functions (65) and adhesion and migration of mast cells (66). In addition to the innate immune system, 3BP2 is required for B cell proliferation and cell survival following cross-linking of the BCR through SYK phosphorylation (67). 3BP2 is also part of a signaling complex of the TCR with LCK, ZAP-70 and VAV that is induced by CD28 co-stimulation, leading to proliferation and differentiation of T cells since 3BP2-deficient CD8+ T cells exhibit a proliferation defect (68). In an animal model of rheumatoid arthritis (RA), 3BP2 deficiency reduced induction of arthritis and bone erosion through reduction of autoantibody production (69), while an increased level of 3BP2 caused exacerbation of bone loss with increased osteoclast formation in an arthritis mouse model (70), indicating that a high expression level of 3BP2 might be a pathogenesis of RA. In addition to 3BP2, the chondrogenic master transcription factor SOX9 is ubiquitylated by another E3-ligase that is mediated by Tankyrase, while Tankyrase inhibitors ameliorate osteoarthritis in mice (71).

These studies have thus shown that dysregulation of E3-ligases-mediated ubiquitylation causes abnormalities in skeletal formation and the osteoimmune system that are associated with a pathogenesis of human autoimmune/autoinflammatory disorders (**Figure 2**). E3-ligases and their substrates could be therapeutic targets for these disorders, and some of them are in preclinical or clinical trials, especially for cancer (72, 73). On the contrary, our recent study showed that RNF146-deficient myeloid cells are highly active for the production of inflammatory cytokines through elevation of 3BP2, indicating that tissue-specific inhibitors or enhancers of E3-ubiquitin ligases or their substrates should be established to avoid unexpected side effects (74). Additionally, these genetic studies have partially uncovered the association between E3-ligases and inflammatory human disorders. Further studies in humans are thus required in order to provide new genetic evidence of other ligases, including NEDD4-2 (75), HUWE1 (76), c-MIR (77), Cullin3 (78), FBW7 (79–82), MARCH1 (83–88), MURF1 (89–92), RNF90 (93–95), SAG (96), Hrd1 (97), Peli1 (98, 99), TRIM (100–102) and MYCBP2 (103), that have been shown to be associated with the osteoimmune system in previous *in vitro* and *in vivo* mouse studies.

## Author contributions

YM: conceptualization and guidance. YA and YM: writing the original draft. JW and RR: proofreading. All authors contributed to the article and approved the submitted version.
